# Nuclear RNA: a transcription-dependent regulator of chromatin structure

**DOI:** 10.1042/BST20230787

**Published:** 2024-07-31

**Authors:** Jon Stocks, Nick Gilbert

**Affiliations:** Medical Research Council Human Genetics Unit, Institute of Genetics and Cancer, University of Edinburgh, Edinburgh, U.K.

**Keywords:** chromatin, nuclear proteins, RNA, transcription

## Abstract

Although the majority of RNAs are retained in the nucleus, their significance is often overlooked. However, it is now becoming clear that nuclear RNA forms a dynamic structure through interacting with various proteins that can influence the three-dimensional structure of chromatin. We review the emerging evidence for a nuclear RNA mesh or gel, highlighting the interplay between DNA, RNA and RNA-binding proteins (RBPs), and assessing the critical role of protein and RNA in governing chromatin architecture. We also discuss a proposed role for the formation and regulation of the nuclear gel in transcriptional control. We suggest that it may concentrate the transcriptional machinery either by direct binding or inducing RBPs to form microphase condensates, nanometre sized membraneless structures with distinct properties to the surrounding medium and an enrichment of particular macromolecules.

## Introduction

**Figure 1.**
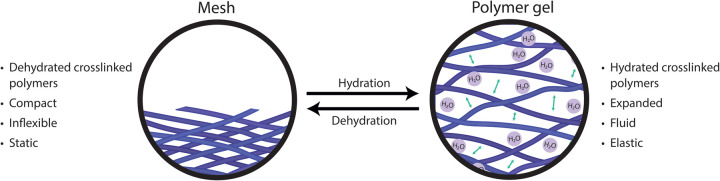
Relationship between mesh and gel structures. A gel can be described as a semi-solid that can range in properties depending on its composition. Structurally, gels are defined as substantially dilute cross-linked systems, and therefore when dehydrated the underlying mesh structure remains. Biophysically, gels exhibit no flow at steady state, but the aqueous phase can diffuse through the system.

The nucleus of mammalian cells is a membrane bound complex containing genomic DNA with RNA and proteins, enabling the protection and replication of DNA and expression of genes. High salt extractions of nuclei reveal a rigid filamentous structure of insoluble proteins and RNA, coined the ‘nuclear matrix’, that was thought essential for cell function through maintaining the shape of the nucleus and regulating gene expression [1–3]. More recently, it is believed that the harsh preparations used to visualise the nuclear matrix are not representative of *in vivo* structures and similar networks have been challenging to identify through microscopy of matrix proteins [4]. Moreover, chromatin, the complex of DNA and associated proteins in the nucleus, has since been thought to dynamically change states rather than being in a single fixed conformation. RNA and RNA-binding proteins (RBPs) are believed to modify chromatin structure, but instead of forming a static, stable structure, RNA and RBPs transiently interact with DNA/chromatin and collaborate to form a mesh-like structure that influences the biophysical properties of chromatin [5,6]. The relationship between a mesh-like structure and gel depends on their hydration state (Figure 1). Typically, a gel is comprised of cross-linked hydrated polymers that form an expanded semi-solid material with properties between a liquid and a solid, allowing it to retain its form yet have flexible and elastic qualities [7]. If dehydrated the gel would shrink leaving a compact structure comprised of cross-linked polymers that form a mesh. In the nucleus, polymers of RNA interact with various RBPs, to create a mesh-like structure, that when hydrated has gel-like properties that are influenced by the constituent proteins and RNA. Nuclear proteins that bind RNA, including SAF-A (or HNRNPU), TDP-43 and MATR3, largely identified from early ‘nuclear matrix’ preparations, form a scaffold that influences chromatin structure [8–10]. Modification of the physical properties and components of gel has been shown to result in changes in chromatin structure and influence multiple nuclear processes such as transcriptional regulation, RNA processing, and the DNA damage response (DDR) [11–13].

Protein components of the nuclear scaffold often have intrinsically disordered regions (IDRs) which have the ability to undergo phase separation, the self-organisation and concentration of molecules into membraneless liquid or gel-like condensates with distinct physical properties to the surrounding medium [14]. These condensates have been associated with super-enhancers as well as abnormal condensates being identified in neurons of patients with neurodegenerative conditions [15,16]. This suggests that phase separation of RBPs plays a key role in the regulation of chromatin structure and transcription. On top of this, aberrant phase separation, differences in the size, localisation, number or composition of condensates, can alter biological processes and are associated with neurodegenerative diseases such as Alzheimer's disease and amyotrophic lateral sclerosis (ALS) [17]. Here, we review recent studies revealing the presence of a complex, dynamic RNA gel that regulates chromatin structure. Moreover, we highlight evidence for the importance of establishing a microenvironment for transcription and discuss recent investigations into the mechanisms behind transcriptional regulation through RNA.

## Composition of the gel

Messenger RNA (mRNA) is produced through capping the 5′ end of the pre-mRNA with a 7-methylguanosine cap, the pre-mRNA is then spliced, the 3′ end is cleaved and a poly(A) tail is added [18]. The processed RNAs are co-transcriptionally packaged into messenger ribonucleoprotein (mRNP) complexes and then translocated through nuclear pore complexes where the mRNPs are released into the cytoplasm for translation [19]. However, <5% of the total amount of RNA produced by RNA polymerase II is exported from the nucleus, with ∼70% of pre-mRNA molecules not being polyadenylated and retained in the nucleus. Most of these transcripts, including many long non-coding RNAs (lncRNAs), intronic, intergenic, repeat rich and antisense RNAs have no known individual roles [20]. These RNA species instead interact with an array of RBPs which together influence chromatin structure, transcription and RNA processing. The majority of RBPs interact with chromatin, either directly or via RNA that is being transcribed or is associated with the chromatin, with an enrichment at regions of open chromatin associated with transcribed genes [21,22]. RBPs are enriched at promoters and colocalise with transcription factors (TFs), however, different RBPs show a preference for different promoters with almost all promoters associating with at least one RBP [22]. The activity of the promoter appears to be linked to the RBPs that associate with it, for example, HNRNPK is largely associated with transcriptionally repressed regions, while some RBPs associate with bivalent promoters, and differ between cell types, either due to different roles or because they are more likely to have different associations depending on gene activity [22]. Whilst the specific actions and cooperativity of RBPs is not currently known, RBPs being found at almost all active genes suggests that they play a key role in the process of transcription and might be recruited in a way that can provide some specificity.

A recent flurry of techniques for investigating RNA-chromatin and RNA-RNA interactions have identified that nascent transcripts and most non-coding RNAs (ncRNAs) are retained near to the site of their transcription [23–28]. Chromatin associated RNA (caRNA), RNA that directly or indirectly associates with chromatin, forms interactions with proximal DNA and RNA with an enrichment of interactions at the promoters and enhancers of active genes. The level of RNA attachment correlates with active histone modifications and transcription, these interactions can be cell type specific and occur hundreds of kb from the site of the site of transcription [23,24]. There are regions of highly concentrated ncRNA, such as NEAT1 or MALAT1, in part localised by RBPs, that enable regulation of proximal genes through regulating chromatin structure, RNA processing and transcription [27]. It is common for both RBPs to be important in determining the localisation of the RNA and RNA to be key to localising RBPs, removal of either a specific RBP or RNA results in mislocalisation of the other and compaction of gene rich regions [29,30]. After knockdown of RBPs RNA can freely diffuse away from its site of transcription, suggesting that RBPs and RNA both act as anchors influencing the localisation of the other.

The properties of RNA including its sequence, length, charge, repeats, modifications and the ability to be quickly produced and degraded enable differences in protein, RNA and DNA interactions and the strength of the interactions. Repeat rich RNA, produced from short and long interspersed nuclear elements (SINEs and LINEs), retrotransposable elements making up over a third of the human genome, is found enriched at transcribed loci and tend to be relatively stable [31,32]. Repeat rich and nascent RNA makes up a significant proportion of the RNA associated with scaffold proteins, including SAF-A and MATR3 [30,33,34].

A significant proportion of the RNA comprising the gel is intronic, indicating that splicing may act to influence chromatin architecture through providing scaffolding RNA as well as to remove introns from coding transcripts [32]. Long, highly expressed genes have been identified to form transcription loops in which the gene extends away from the chromosome [35]. The nascent RNA that is being transcribed associates with RBPs to form bulky ribonucleoproteins (RNPs) that increases the stiffness of the loops, the extended formations and rigidity may provide a mechanism to increase the efficiency of transcription of these genes. Surprisingly, inhibition of splicing resulted in an increase in the size of these loops, this is likely due to an increase in the size of the RNPs that affect the stiffness of the gene [35]. It is possible that these events occur at a smaller scale, with retention of introns in nascent transcripts modifying the 3D structure of the gene during transcription. Further to this, with RBPs such as SAF-A shown to regulate splicing, it is possible that RBPs influence on splicing also acts to affect the local chromatin structure [36].

Other ncRNAs making up the gel include antisense transcripts, this class of RNA is found at more than 30% of human genes, where it has been linked to transcriptional regulation, with the knockdown of several antisense lncRNAs also reducing the transcription of associated protein-coding genes [37,38]. Antisense RNA may be important by providing elevated RNA levels at gene promoters to provide greater feedback to affect the local chromatin environment and hence transcription. The antisense ncRNA *Evx1as* is coexpressed alongside the protein-coding gene *EVX1*; the antisense transcripts attach to their own site of transcription and associate with the transcriptional co-activator Mediator enabling a fine tuning of transcription [39]. Antisense transcripts have also been noted to have specific roles in regulating transcription of their associated protein-coding gene through mediating chromatin modifications, especially promoting DNA methylation of CpG island promoters [40]. The diverse roles and effects of antisense transcripts highlights how more research must be carried out to understand their cross-talk and regulation of neighbouring genes.

The cycle of transcription and turnover of transcripts means that the concentration of RNA around genes allows the properties of the gel to be rapidly modified in response to the local requirement at any one time. Modifications of the RNA can affect the RNA's stability and associations, for example, methylated enhancer RNA (eRNA) has been found to be more stable enabling a longer lasting scaffold to be produced [41,42]. The m6A (N6-methyladenosine) reader YTHDC1 affects transcription through binding methylated RNA and recruiting transcription regulators, while methylation of nascent transcripts enables YTHDC1 to be enriched at active genes where it promotes the formation of foci with the transcriptional co-activator BRD4 [42]. However, YTHDC1 has also been oberved to be involved in recruitment of the exosome which can degrade a subset of methylated caRNAs, and in turn reduce the methylation of these caRNAs to promote an open chromatin state and transcription [43].

The physical characteristics of RNA establish its suitability to tailor the properties of the nuclear gel for specific genes through differences in its associations and stability, however, the differences in these properties have on chromatin structure and transcription suggest that the results are context-dependent and so more work is required to understand the specific mechanisms involved.

## RNA and chromatin structure

Transcriptionally active, gene-rich regions of the genome are enriched in RNA and scaffold proteins and at Mb scales tend to be more open than inactive, gene-poor regions [10,44,45]. Knockdown of the majority of investigated RBPs result in changes in both chromatin architecture and transcription, however, it is hard to discriminate whether one of these changes is responsible for the other [22]. This is the case for SAF-A (Scaffold Attachment Factor A; also known as HNRNPU), a nuclear RBP associated with processes including chromatin organisation, transcription and RNA processing. Transcription inhibition or SAF-A knockdown results in compaction of open gene rich regions to an inactive like state [10,33,46]. This gel-dependent chromatin decompaction, could be achieved in a variety of ways: as a scaffold to localise chromatin modifiers or by altering the properties of the microenvironment around the chromatin fibre.

Matrin 3 (MATR3) is an abundant nuclear protein containing both RNA and DNA binding domains as well as extensive disordered regions [47]. MATR3 was originally identified as a constituent of the nuclear matrix and is now considered important for transcriptional regulation, possibly through forming a dynamic protein network that influences chromatin architecture [48]. MATR3 colocalises with H3K27me3, whilst MATR3 knockdown results in a redistribution of H3K27me3 and increased accessibility to previously repressed regions [30]. This is thought to be due to the MATR3-RNA network restricting chromatin movement and direct interactions between MATR3 and RNAs with Polycomb Group Proteins (PcGs) responsible for the histone methylation, and suggests that RBPs, and therefore RNA, are required to localise factors involved in modifying histones and influencing chromatin architecture.

RNA is able to influence the compaction of chromatin directly through altering histone interactions with DNA in a sequence independent manner. RNA is strongly negatively charged, the dense negative charges at sites of transcription neutralise histone tails preventing histone self-association and histone-DNA interactions, therefore causing chromatin to adopt a more open conformation [49]. On top of this, RNA has a stronger association with chromatin than linker histone 1 (H1), the protein involved in binding linker DNA and condensing chromatin into higher order fibres, which allows caRNA to deplete regions of chromatin of H1 resulting in a less compact structure [50].

An RNA gel has also been suggested to influence chromatin structure by regulating topologically associated domains (TADs), segments of the genome that are thought to form micro-environments by promoting interactions between regulatory elements. CTCF and cohesin are DNA binding proteins involved in forming the boundaries of TADs, but interestingly, it has been proposed that RNA associates with these proteins to influence the positioning and strength of TAD boundaries [51,52]. The mechanism is unclear, but RNA is enriched at TAD boundaries, especially at promoters, which may be due to an enrichment of RBPs at these sites [53,54]. Transcription inhibition or deletion of CTCF's RNA binding ability decreases CTCF's ability to self-associate and bind chromatin, especially at promoters and transcription start sites, suggesting that RNA influences DNA looping through its association with CTCF [55,56]. RNA promotes DNA looping which may indirectly influence gene expression, however, RNA binding mutants of CTCF reduces the strength of almost half of TADs, demonstrating the importance of RNA in influencing chromatin structure is dependent on multiple factors. Cohesin can also bind to RNA through its SA1 and SA2 subunits, often at sites enriched in R-loops, three-stranded nucleic acid structures of a DNA:RNA hybrid and a displaced ssDNA loop [52]. In these locations it has been proposed that R-loops enable the complex to efficiently identify transcribed regions of the genome allowing formation and maintenance of TADs at active gene loci. Altogether, this suggests that RNA enhances structural features at its gene acting as a messenger in the feedback of transcription onto structural proteins, but rather than acting as a determinant of chromatin structure RNA is able to fine tune protein associations allowing a greater effect at genes that have a variable level of expression.

## RNA interactions and transcription

It is plausible that the RNA that forms the scaffold at genes directly interacts with TFs resulting in an increased local concentration of transcription machinery, on a gene specific basis. Oksuz et al. [57] discovered that as well as binding DNA and other proteins at least half of TFs also bind RNA originating proximal to their target genes. Most of these TFs were found to have a motif of conserved basic residues necessary for RNA-binding nearby their DNA-binding domain, similar to the arginine rich motif RNA-binding domain of the HIV Tat transactivator. Removal of the RNA binding domains of TFs resulted in a decrease in both the binding of TFs to DNA and the level of transcription, likely as a result of a decreased fraction associated with chromatin. Although this may be in part due to the RNA binding domain having some DNA binding ability, the interaction domain exhibits stronger binding to RNA and in some cases, such as for SOX2, deletion of the RNA binding domain shows no change in DNA binding *in vitro*, yet results in developmental defects in zebrafish. Specific TFs such as YY1 have also been shown to bind DNA and RNA independently [58,59]. RNA guides and enhances YY1 DNA binding, enabling it to efficiently modify chromatin architecture and transcription. Moreover, the transcriptional co-activators CBP and BRD4 have been noted to be localised to enhancers through eRNA which also stimulates their histone acetyltransferase activity promoting further transcription [60,61]. Altogether, this suggests that the RNA scaffold retains TFs at their target loci, enabling efficient transcriptional regulation with a degree of specificity provided by RNA. There may also be variable amounts of different RBPs comprising the scaffold at different genes driven through interactions with DNA, RNA and proteins, which could enable differences in localisation and turnover of RNA and so influencing chromatin structure and transcription.

RNA has not only been found to promote the localisation of transcriptional activators but has been shown to antagonise factors involved in repression of transcription. Skalska et al. [62] recognised that many factors associated with transcription repression, including transcriptional regulators, chromatin modifiers, DNA methyltransferases, OCT4 and cohesin, associate with nascent transcripts. As nascent RNA directly interacts with transcriptional repressors this indicates that the expression of active genes supresses the activity and binding of these inhibitory factors. These factors are also able to act on inactive genes or active genes upon transcription inhibition, promoting a repressed state due to the lack of RNA that normally inhibits their localisation. Polycomb Repressive Complex 2 (PRC2), the factor responsible for trimethylating H3K27, inducing transcriptional silencing, has also been found to associate with RNA. Despite being a transcriptional repressor, PRC2 is found at active genes where it associates with nascent transcripts, and promotes a structural change in the complex so that PRC2 dimerises enclosing the domain involved in binding DNA and the histone H3 tail [63]. The association with chromatin is also antagonised by RNA through outcompeting DNA to interact with PRC2, this allows transcription to inhibit the deposition of repressive histone modifications at the gene [64]. Specificity for PRC2 has been found to be provided through interacting with G4 RNA quadruplexes, guanine rich sequences folded into non-canonical secondary structures [65]. PRC2 shows preferential binding to G4 quadruplexes within nascent RNA, tethering G-tract RNAs to a gene decreases the chromatin occupancy of PRC2 and the level of H3K27 methylation. This suggests G4 quadruplexes are sequence specific interaction partners of PRC2 that bind and displace PRC2 from chromatin upon gene activation, resulting in a change in histone medications and, at least for some genes, a change in transcription.

These studies indicate that RNA can associate with TFs and chromatin modifiers, in some cases in a sequence-specific manner, enabling the output of a gene to provide feedback to localise, activate or inhibit proteins that modify its activity. With many factors which bind to RNA still being identified, future work on TFs is likely to delve deeper into the mechanisms of how RNA is able to concentrate specific regulatory components and whether this is achieved through sequence specific interactions with RNA or whether DNA and RBPs act to recruit these factors and RNA is involved in retaining them and fine-tuning the level of transcription.

## Phase separation

Most RBPs contain IDRs that lack a stable 3D structure; they are often enriched in abundant charged and polar amino acids with few bulky hydrophobic amino acids [66]. IDRs provide proteins with a propensity to self-associate and produce phase condensates, liquid-like droplets of concentrated proteins, RNA and other molecules. However, the high concentration of RNA in the nucleus often reduces interactions between these domains, through charge interreference, allowing RBPs to be largely soluble in the nucleus [67,68]. Yet, in the presence of specific RNA species some RBPs phase separate to form condensates such as paraspeckles, nucleoli and stress granules, as well as abnormal phase separation occurring in neurons of patients with neurodegenerative conditions [16]. In these situations phase condensates likely arise from sequence specific interactions with RNA driving a high local concentration of certain RBPs. The RNA binding capacity of proteins in the RNA-protein scaffold also influences the propensity for the occurrence of phase separation. G3BP is a protein involved in stress granule formation, an acidic region of the protein next to the IDR prevents RNA association and phase separation [69]. However, *in vitro* experiments demonstrate that with sufficient RNA the RNA association can outcompete the protein auto-inhibitory interaction enabling protein clustering and inducing phase separation. The frequency of IDRs in RBPs suggests that this may be a common mechanism with the different inhibitory effects of the IDR and the affinities for RNA dictating the likelihood of phase separation occurring at different RNA concentrations and strengths of association [70]. It is though necessary to investigate other RBPs and their RNA associations to understand if this is a common phenomenon and whether it plays a physiological role.

RBPs can influence the properties of RNA to induce the formation of coacervates, condensates formed through interactions of oppositely charged molecules [71]. Formation of condensates is promoted by highly negatively charged RNA lacking a secondary structure which can be affected by protein interactions, furthermore, protein modifications that alter the charge balance, such as phosphorylation, have been suggested to provide a possible mechanism to regulate phase separation [71]. Further to this, multiphase coacervates, condensates with varying local viscosities that partition different proteins and nucleic acids, are produced through varying charge densities and RNA-RBP interactions [72,73]. Secondary structures of RNA and interactions with RBPs influence the layer in the coacervates in which the RNA species are present. RNA base pairing decouples the dynamics of RNA and RBPs and results in the formation of more rigid open networks giving different regions of the same condensates different properties [72]. Multiphase coacervates could provide a potential mechanism to regulate transcription by concentrating transcription machinery in different phases and proximity to a gene [15,74].

Aberrant phase separation and aggregation of RBPs in neurodegenerative disorders has been associated with mutations in the RBPs changing their RNA binding characteristics, protein structure or post translational modifications, any of these changes can result in aggregation or mislocalisation of RBPs which goes on to affect transcription and cell survival [16,75]. TDP-43 and FUS aggregates are commonly observed in neurons of patients with ALS and frontotemporal dementia (FTD) [76]. TDP-43 in these aggregates is often atypically ubiquitinated, phosphorylated, contain phosphomimetic mutations or truncations, all of these modifications increase the protein's propensity to phase separate [77,78]. Similarly, in FTD, FUS has a hypomethylated RGG domain which increases its ability to phase separate, however, in ALS, the RGG domain is methylated reducing its ability to bind to its nuclear import receptor resulting in its accumulation in the cytoplasm and being recruited to stress granules. TDP-43 and FUS have also been implicated in regulation of transcription, splicing, RNA stability, RNA localisation and mRNA export. Therefore, any change in their localisation and activity is likely to have significant effects on the cell, on top of this, ALS linked TDP-43 mutations show an impairment of DNA repair pathways leading to cell death and TDP-43 accumulating in mitochondria resulting in increased inflammation [79,80].

Phase separation can also be induced through mutations in RNA; modifications to coding and ncRNA in which short repeats are added to the transcripts, depending on the sequence, and the number of repeats, can result in intermolecular base-pairing that promote the RNA accumulation resulting in phase separation similar to that seen in protein aggregation disorders [81]. Moreover, RNA with repeats of CAG and sufficient adenosine methylation can directly associate with TDP-43 promoting it to mis-localise to the cytoplasm and be partitioned into stress granules. Therefore mutations in both RNA and RBPs can cause irregular phase separation that may alter neuronal cell behaviour [82].

RNA induced phase separation of proteins has been shown to have roles in stress responses, RNA metabolism, DNA damage response (DDR) and promoting the activity of super-enhancers [12,74,83,84]. Super enhancers, clusters of enhancers occupied with a high density of transcriptional activators that drive elevated levels of transcription, have been shown to colocalise with condensates, consequently it has been proposed that the high density of transcriptional activators at super-enhancers drives condensate formation [74]. RBP-RNA gels recruit TFs, RNA Polymerase II and the mediator complex, high local concentrations of these factors promotes protein-protein interactions via IDRs resulting in phase separation [85,86]. These condensates increase the concentration of factors involved in transcriptional regulation, even those not directly involved in forming the condensate, such as kinases, that are able to induce phase condensation and recruit RNAPII. This process results in a condensate with concentrated enzyme and substrate, promoting hyperphosphorylation of the C terminal domain of RNAPII enabling transcription elongation [87,88]. Comparison of the motion of TADs to simulated data suggests that they can also be organised as weak-gel droplets, in which the TAD is reversibly bonded with short-lived cross-links [89]. Changing the level of cross-linking determines the physical state, with reduced cross-linking resulting in gel dissolution and increased cross-linking promoting phase separation. Proximity to a phase transition boundary subsequently enables TFs to induce a rapid rearrangement of chromatin and alter the levels of the transcription machinery and therefore affect the level of transcription.

To understand how RNA may regulate its own transcription, Henninger et al. [68] used *in vitro* experiments to investigate how changes in the levels of RNA whilst a gene is transcribed affects the formation and stability of condensates, positing a way for RNA to provide feedback on transcription. Low levels of RNA promote MED1 phase separation with droplet size peaking at ∼4 µm^2^ in the presence 100 nM RNA (and 200 nM mediator complex) whilst higher RNA concentrations result in dissolution of condensates and reduce the level of transcription in *in vitro* transcription assays. Moreover, inhibition of transcription elongation *in vivo* results in longer lived condensates. Collectively, this suggests that when transcription is initiated nascent transcripts stimulate the formation of condensates with an average volume of 0.02 µm^3^, however, during elongation the high concentration of local negatively charged RNA alters electrostatic interactions resulting in the condensate dissolving and a reduction in the concentration of transcriptional activators. Transcription is thought to occur in periodic bursts, which the charge of RNA could account for; as transcription persists there is a build-up of negatively charged transcripts that could dissolve the condensate resulting in a loss of transcription machinery and a pause in transcription until the machinery is reassembled, essentially providing a feedback mechanism.

**Figure 2.**
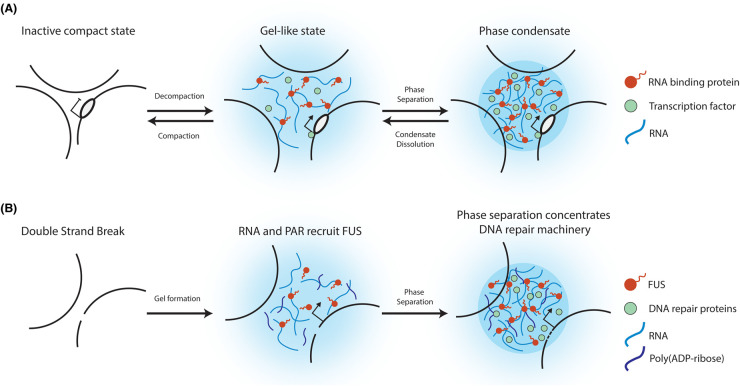
Phase separation of RBPs at sites of transcription and DNA damage. (**A**) Transcription initiation triggers the accumulation of nascent RNAs (blue) in the vicinity of active genes. RNAs interact with RBPs (red), such as SAF-A, to create a gel-like state and forming microphase concentrates through the interaction of IDRs of the RBPs. This microenvironment is enriched in negative charges and can trap polymerases, transcription factors and other proteins (green) to facilitate and promote transcription. (**B**) DNA double strand breaks results in the recruitment of transcription machinery, resulting in the accumulation of RNA (light blue) and the production of poly(ADP-ribose) (PAR) (dark blue). This results in the recruitment of FUS (red) which phase separates and concentrates DNA repair machinery (green) at sites of damage.

The DDR can modify proteins associated with RNA metabolism of double strand breaks (DSBs) as well as induce the transcription of RNA from these sites [90–92]. Pessina et al. showed that inducing DSBs results in the recruitment of the RNA Polymerase II preinitation complex, as well as MED1 and CDK9, at these sites resulting in immediate transcription [93]. They suggest that the large quantity of RNA produced promotes the formation of the observed phase condensates which enable the concentration and localisation of DNA repair machinery. The localisation of RNA suggests that RBPs may be involved in this process as well, especially as many RBPs have been noted to be involved in recognising and repairing DNA damage [94]. For example, the RBP FUS is important for inducing phase separation and enabling the recruitment of DDR factors, the identification of transcription being an early event after DNA damage raises that possibility that the role of the RNA is to recruit FUS, the RNA and FUS can then co-operate to form condensates to concentrate DDR machinery enabling efficient repair (Figure 2A) [12]. As well as RNA, poly(ADP-ribose) (PAR), an RNA-like polymer, is produced upon DNA damage and drives FUS to phase separate [95]. Whilst FUS stably interacts with RNA, the interaction between FUS and PAR is transient allowing rapid condensate formation. Moreover, the ratio of PAR and RNA influnces the size and dynamics of FUS condensates, illustrating how different molecules collaborate to drive efficient localisation of repair machinery (Figure 2B).

Phase separation of RBPs may be a common mechanism in the regulation of transcription. However, the scale of condensates may differ for different levels of transcription, raising the possibility of microphase separation regulating transcription at genes that are not as highly expressed as those regulated by super-enhancers [96]. Regardless of this, it is clear that RBPs play a significant role in the regulation of chromatin structure and gene transcription. The RNA-protein gel needs to be investigated further in order to understand the extent it plays a role in the dynamic regulation of transcription. This could include rapid changes in the properties and composition of the RNA-protein gel upon transcription activation that can enable further transcription via modulating the concentration of the transcription machinery and by providing an accessible environment for decompacting gene loci. Neuronal immediate-early genes are a set of genes involved in key roles in synaptic processes and are dramatically and transiently up-regulated, RBPs may be critical in enabling these genes to meet both extremes of expression providing a possible explanation of the association of RBPs with neurodevelopmental conditions [97,98]. The RNA produced at these genes could provide positive feedback for transcription rapidly recruiting RBPs, which in turn establish a transcriptionally permissive environment enabling massive levels of transcription to be achieved. In the RBP-associated neurological conditions, mutations in these proteins may result in the inability to successfully form the required microenvironment, resulting in less significantly up-regulated gene expression impacting development and cell survival.

## Concluding remarks

Early studies into the nuclear architecture identified the presence of a rigid ‘nuclear matrix’ structure that was thought to regulate chromatin structure and gene expression. Despite this having since been disproved, a newer model has since been proposed in which RNA and RBPs transiently interact to form a hydrated mesh or gel. The components of this gel have been idenitified to modify chromatin structure and have been associated with multiple nuclear processes including transcription, RNA processing and DDR. These activities are likely achieved through RNA and RBPs forming specific interactions and promoting phase separation that concentrates factors at specific loci. As our knowledge of the formation and properties of condensates and the cooperation between different RNA species and RBPs continues to grow, so too will our understanding of how their activities are regulated and achieved as well as how abnormalities in these systems can result in neurodevelopmental conditions.

## Perspectives

- caRNA and RBPs co-operate to produce a transcriptionally responsive gel that is involved in regulating chromatin structure.
- Increasing evidence suggests that an RNA-protein structure plays a role in transcriptional regulation. This is achieved by RNA modulating the localisation and activity of proteins as well as promoting phase separation that enables proteins to be highly concentrated at the site of transcription.
- Future work should focus on the mechanisms linking RNA to transcriptional regulation, especially, at genes not associated with super-enhancers. This may require investigating interactions between scaffold proteins to discover if different proteins regulate different genes, act in different cell types, or act on the same genes either cooperatively or antagonistically.

## Open Access

Open access for this article was enabled by the participation of University of Edinburgh in an all-inclusive *Read & Publish* agreement with Portland Press and the Biochemical Society under a transformative agreement with JISC.

## Abbreviations

ALS: amyotrophic lateral sclerosis
caRNA: chromatin associated RNA
DSB: double strand break
eRNA: enhancer RNA
FTD: frontotemporal dementia
IDR: intrinsically disordered region
lncRNA: long non-coding RNA
mRNA: messenger RNA
mRNP: messenger ribonucleoprotein
ncRNA: non-coding RNA
PRC2: Polycomb Repressive Complex 2
RBP: RNA-binding protein
RNP: ribonucleoprotein
TAD: topologically associated domain
TF: transcription factor
